# Proxy responses to ICECAP-A: Exploring variation across multiple proxy assessments of capability well-being for the same individuals

**DOI:** 10.1371/journal.pone.0236584

**Published:** 2020-07-28

**Authors:** Philip Kinghorn, Nafsika Afentou

**Affiliations:** Health Economics Unit, Institute of Applied Health Research, University of Birmingham, Birmingham, United Kingdom; Washington University, St. Louis, UNITED STATES

## Abstract

**Background:**

The ICECAP capability measures are increasingly being used to capture the impact of health and social care interventions on well-being. In cases where the recipient of an intervention is highly vulnerable, proxy completion may be necessary. This study adds to the limited existing evidence on proxy completion of ICECAP-A specifically and adopts the novel approach of investigating multiple proxy responses for the same four (hypothetical) individuals.

**Methods:**

62 members of the public who were participating in a series of one day deliberative workshops on public health and social care completed ICECAP-A on behalf of four hypothetical individuals, described in vignettes. Quantitative analysis explored the range of proxy responses for each of the four hypothetical individuals, and any possible correlation between participants’ own characteristics and their proxy responses. Participants discussed their proxy responses after completing the task; this discussion was audio recorded and analysed using Framework Analysis.

**Results:**

Wide variation in ICECAP-A scores was observed across proxy respondents for each hypothetical individual. Participants’ demographic characteristics and own well-being do not appear to have systematically influenced proxy responses. Qualitative analysis suggests two principal approaches (or perspectives) were adopted by participants: Empathetic (adopting the perspective of the ‘subject’) and factual (a factual assessment of the subject’s well-being). Participants also drew on their own experiences to varying degrees. There were differing interpretations of the Independence attribute on ICECAP-A and some evidence that participants’ ideas of what constituted achievement were context (including life-stage and condition/health) specific.

**Conclusions:**

The factual versus empathetic approaches identified from qualitative analysis in this study match to the concept of a proxy-proxy versus proxy-patient perspective, previously outlined in the literature. Researchers should consider specifying which perspective proxy raters should adopt. Findings also suggest proxy responses can be influenced by external points of reference and interpretation of measure attributes.

## Introduction

The ICECAP-A (ICEpop CAPability measure for Adults) is a measure of capability well-being, intended for use in economic evaluation [1]. The measure was developed in the UK, but has also been used in other English speaking countries and across Europe [2].

The measure is distinct from those typically used in health economics, both because of its conceptual alignment to the capability approach of Amartya Sen [3, 4] and the breadth of its evaluative space–well-being in a broad sense, rather than health-related quality of life. The capability approach relies on a distinction between functionings and capabilities (achieved or observed outcomes versus the ‘real’ opportunity that a person has to be or do the things they have reason to value) [3]. ICECAP-A seeks to elicit information on capability by phrasing questions in terms of “I am able to…” and “I can have” [1]. The measure covers five areas of life (or five key capabilities): Stability (feeling settled and secure); Attachment (love, friendship and support); Autonomy (being independent); Achievement (achievement and progress); and Enjoyment (enjoyment and pleasure). There is one question relating to each of the five capabilities.

There are four response options available for each of the five questions [1] and responses are coded (for the purpose of data entry) such that level 1 is the worst response option (for example, “I am unable…” or “I cannot have…”) and level 4 is the best response option (for example, “I can have all/a lot of…”). A value set (or scoring system) exists for ICECAP-A [5], such that a respondent who selects level 1 for all five questions (giving a profile of: 1,1,1,1,1) will have a score of 0 and a respondent who selects the top level for each question (giving a profile of 4,4,4,4,4) will have a score of 1. A score of zero is interpreted as “no capability” and a score of one is interpreted as “full capability” [5].

ICECAP-A is one of four ICECAP measures, which also include the ICECAP-O for older people [6], the ICECAP-SCM (Supportive Care Measure) [7] and the ICECAP-CPM (Close Person Measure), a measure for assessing the impact of end of life care on those close to the patient [8].

Al-Janabi *et al* [9] note that, in the context of human development (where the capability approach was first developed), functionings have frequently been used as a proxy for capability (often with reliance on interrogation of existing datasets), whereas in health research conducted in high income countries, there is commonly an emphasis on primary data collection, relying on self-reporting. Hence, although ICECAP-A has generally been found to have good construct validity [10], Al-Janabi *et al* conducted qualitative research to explore whether respondents comprehended the concept of capability and responded appropriately [9]. It was found that a majority of informants participating in think-aloud interviews were able to comprehend questions phrased in terms of capability and felt able to judge their own capability well-being. Indeed, in some cases, informants confirmed that their capability was greater than their level of functioning [9], for example where family circumstances or a relationship constrained their ability to be completely independent. Al-Janabi *et al*. relate this constraint to Nussbaum’s notion of ‘combined capability’ [9, 11], the level of capability reflecting external conditions.

Whilst the results reported by Al-Janabi *et al*. are promising, there will be cases where a patient or recipient of social care (sometimes referred to as long-term care) will be too ill and/or too vulnerable to self-report their own capability well-being, and in these cases it may be necessary to elicit proxy responses. For example, Makai *et al*. relied on staff from nursing homes within Germany [12] and The Netherlands [13] to report the well-being of residents using ICECAP-O. Bailey *et al*. elicited proxy responses using ICECAP-A from close persons and care staff for patients receiving hospice care [14].

Well-being, in general, is ideally assessed by self-report and there are documented challenges associated with proxy completion [15]. A systematic review by Rand and Caiels, covering the period 2004 to 2014 [15], found that whilst a majority of studies reported proxy respondents rating quality of life lower than self-reported quality of life, the direction of difference was not consistent across all identified research studies, and was associated with: methodology (measurement properties of the instrument); the balance of objective/subjective attributes within the measure and the closeness/relationship between the proxy and the subject (pp1-2).

There is also evidence that the cognitive process (or perspective) adopted by the proxy may influence the difference between proxy and self-ratings [15]; Pickard and Knight [16] set out two perspectives from which the proxy can assess the health of a patient. The first involves the proxy assessing the patient in terms of how they think the patient would respond, and is labelled the ‘proxy-patient’ perspective. The second perspective is ‘proxy-proxy’, where the proxy responds based upon their own perception of the patient’s health.

Most empirical studies investigating proxy completion report the level of agreement between responses from self-completion and proxy completion, often on measures of health-related quality of life (as per the studies identified by Rand and Caiels). Makai *et al*. instead report correlation between different proxy groups; they found little agreement between proxy responses provided by care staff and family members in the Dutch study, and suggest that these two proxy groups may have different reference points, with care staff referencing one resident’s capability against that of other residents and family referencing the resident’s capability against the resident’s own previous level of capability [13].

The study by Bailey *et al*. involved patients recruited from a UK hospice (along with close persons and care staff) completing ICECAP-A, ICECAP-SCM and EQ-5D within think-aloud interviews [14]. The paper reports error rates relating to comprehension, retrieval, judgement and response. Qualitative analysis revealed that healthcare workers found it easier to complete EQ-5D (as a measure of health functioning) on behalf of patients than they did to proxy using the ICECAP measures (with their focus on broader well-being); there were also fewer errors when healthcare workers completed EQ-5D, in comparison to the ICECAP measures [14].

In summary, proxy respondents are commonly observed to report the subject’s well-being as worse than the subject’s own self-assessment; different proxy respondents may adopt a different reference point or find it easier to report on some aspects of a subject’s well-being than on others, depending upon their relationship with the subject; and the perspective adopted by a proxy respondent (proxy-proxy or proxy-patient) may also influence their proxy responses.

This study is the first to elicit qualitative and quantitative data from multiple proxy respondents for the same four, hypothetical individuals. The aim of the study was not to explore differences between proxy and self-reported responses, but instead to explore variation across multiple proxy responses for the same ‘subject’ individual and attempt to explain any significant variation in proxy responses. Objectives were to: (i) assess the extent of divergence in proxy responses for the same ‘subject’ individual; (ii) investigate whether there was any evidence of proxy responses varying systematically across proxy raters of different socio-demographic groups; and to understand the cognitive process, reported difficulties, struggles and considerations of proxy raters.

## Methods

A series of eight one-day deliberative workshops (involving a total of 62 members of the public) were held across the West Midlands region of the UK, between August and November 2016. The primary objective of the workshops was to elicit a sufficient level of capability well-being (as defined by ICECAP-A), for use in decisions about the allocation of scarce public health and social care resources [17]. The intuition behind eliciting a sufficient level of well-being is that society will not attach additional value to improvements in well-being resulting from social policies beyond what is deemed to be a ‘good enough level’. Practically, this means prioritising improvements in well-being for the worst off in society above improvements for those already in a “comfortable” or “adequate” state of well-being [17].

Deliberative processes involve presenting options and information to participants and encouraging open discussion, in order to elicit informed and considered views. To prompt participants to consider a range of social care and public health services and how these services may affect the lives of those dependent upon them, participants were presented with hypothetical vignettes. Vignettes depicted the needs and circumstances of hypothetical individuals and the services/sources of support which may be available to them. To develop familiarity with the ICECAP-A, workshop participants were asked both to self-complete ICECAP-A and to provide proxy responses on behalf of the ‘subject’ individuals described in the vignettes. Consideration of the vignettes and the completion of ICECAP-A on behalf of the individuals they depict provided the data that is analysed and interpreted in this paper.

### Vignette development

Because the objective was to elicit proxy responses from all workshop participants and facilitate discussion by the group of their shared experience of providing proxy responses for the same ‘subject’ individual, it was necessary for them to base their proxy responses on a vignette, rather than to proxy for somebody that they had a personal connection with. Hypothetical vignettes were selected primarily on the basis that it would be ethically problematic to present information about the lives of real (‘subject’) individuals for discussion and judgement by workshop participants who they had no professional or social connection to. Furthermore, identifying actual service users with a diverse range of needs and circumstances as subjects for the vignettes would potentially have necessitated partnering with multiple gatekeeping organisations.

Hence, a pragmatic approach was adopted whereby hypothetical vignettes were developed by the research team; informed by resources such as the NHS Choices website and the public websites of charity organisations. Feedback on an initial set of six vignettes (in terms of plausibility, factual accuracy and the accuracy of terminology) was obtained from academic colleagues from Nursing, Public Health and Social Work, as well as from the project advisory group (including lay representatives). After amending the vignettes to respond to feedback from academic peers, vignettes were further refined through a series of ‘think-aloud’ interviews with participants recruited from the charity sector. Think aloud interviews assessed comprehension and perceived plausibility of the vignettes and gave an indication of the amount of time needed for participants to read and consider them. Participants in the think-aloud interviews were asked to complete ICECAP-A on behalf of the individuals described within the vignettes and a discussion ensued as to whether they felt that they had sufficient information to do this and whether they had needed to make assumptions. A total of nine think-aloud interviews were conducted with participants recruited via five different organisations.

Four vignettes were *initially* selected for use in the workshops on the basis that they described potential service users (public health and/or social care) with a variety of support needs and representing a variety of ‘life stages’. Vignettes did not specify sex, race or religious beliefs. Vignettes did, however, name specific types of illness. The *initial* vignettes are summarised below:

- Person A: Depression (aged 19 years, living with parents and a sibling and working part-time, having delayed going to university)
- Person B: Planned pregnancy (aged 29, in a happy and stable relationship, slightly over-weight and a smoker, wishing to quit)
- Person C: Multiple Sclerosis (aged 31, recently stopped working due to their health condition and living alone)
- Person D: Dementia (a 68 year old, living alone, but with a son who visits and a neighbour who will also check on them)

The first workshop was used as a pilot workshop and as a result of experience from the pilot workshop one of the vignettes (person C) was changed. This was done because it was deemed that the vignettes over-represented scenarios in which the individual faced serious negative circumstances and constraints. The final set of full vignettes (used in the remaining seven workshops) can be found in the Supporting Information (S1 Appendix); the revised Vignette C is summarised below:

- Person C: Severe visual impairment (aged 47 years, employed, has a partner and a child)

### Workshops & proxy completion

Local authority areas (electoral wards) were purposefully selected to achieve a mix of urban and rural areas, as well as areas of low, mid and high deprivation. Edited electoral registers were obtained from local authorities for the selected electoral wards and members of the public were randomly selected from those electoral registers. Those selected were sent a letter by post inviting them to participate in the research. Those interested in participating returned an expression of interest form, providing information on age, sex and ethnicity. Confirmation letters were then sent out to those expressing interest, followed-up by email and text message reminders. Workshops were held at locations within the selected electoral wards; participants only attended one workshop.

At the workshops, participants were provided with definitions of social care and public health, were informed about the development and purpose of the ICECAP-A and asked to self-complete the ICECAP-A. Participants discussed their initial reaction to the four hypothetical vignettes and were then asked to complete ICECAP-A on behalf of the individuals described in the vignettes. Once all participants had individually considered and recorded their proxy responses on ICECAP-A, they were encouraged to share and discuss their proxy responses, and the reasoning behind their responses. Discussion was audio recorded and transcribed verbatim.

### Ethics

Ethical approval (covering all aspects of the study) was obtained from the Science, Technology, Engineering and Mathematics Ethical Review Committee at the University of Birmingham [ERN_16-0027A]. Informed, written consent was obtained from all participants prior to participation and data collection.

### Analysis

Quantitative analysis was used to explore the following:

- Correlation between participants’ own well-being scores and their proxy responses. It was hypothesised that a person’s own well-being may influence whether they interpret aspects of the vignette more positively or negatively.
- Whether there was any statistically significant difference between the proxy responses of male and female participants, or between the responses of different age groups. It is possible that participants may have found it easier to empathise with individuals from the vignettes in cases where the individual was a similar age.
- Whether individuals providing proxy responses avoid extreme response levels, tending instead to opt for middling levels. This may indicate caution or uncertainty in responses.
- The extent of variation with respect to proxy responses for the same vignette.

Participants’ own ICECAP-A scores and proxy scores for the vignettes were calculated from the UK values published by Flynn *et al* [5]. Correlation between own and proxy scores (at the whole sample level) was assessed visually using scatter plots. Descriptive statistics for ICECAP-A scores are reported and compared after the sample is split by sex and age. *t*-tests were used to test for statistically significant differences in the proxy responses between male and female participants, and Analysis of Variance (ANOVA) to test for differences by age (with the sample split into three age ranges).

The percentage of participants selecting each response level is reported across each of the five attributes on ICECAP-A, for each of the vignettes (S1 Table). There were no *a priori* expectations regarding the ordering of vignettes A, C and D. There was, however, an intuitive expectation that vignettes in which an illness or disability were described as limiting the person’s ability to undertake activities would be ranked below the vignette describing planned pregnancy (i.e. vignette B would have the highest ICECAP-A score).

Framework analysis was used to analyse qualitative data relating to proxy completion of ICECAP-A. Five stages of analysis were followed [18]: (i) familiarisation; (ii) identifying issues and themes (initial coding); (iii) indexing (applying the coding framework systematically to all of the data); (iv) categorisation; (v) interpretation (defining concepts). No preconceived coding or conceptual framework was imposed on the data.

### Reflexivity

Vignettes were drafted by the author (PK), who also conducted the think-aloud interviews as part of the vignette development. PK also facilitated the workshops (together with an administrator and an assistant facilitator). PK is a health economist with experience of undertaking qualitative research (including think-aloud interviews and research involving vulnerable participants). Coding and analysis of transcripts was undertaken by PK and NA, with discussions to agree coding frameworks and then themes. NA is also a health economist, with previous experience of qualitative methods.

## Results

### Sample

3,685 invitation letters were sent to voters within the selected electoral wards and 62 members of the public participated, across the eight workshops. Attendance at each workshop varied between four and 10. Table 1 reports sample characteristics. Older people and females were over-represented in the sample.

**Table 1:** Demographics (Citizens’ workshops).

Characteristic	Total (%)
*Sex*	
Male	24 (38.7%)
Female	38 (61.3%)
*Age ranges*	
Aged 18–24	2 (3.2%)
Aged 25–35	6 (9.7%)
Aged 35–44	3 (4.8%)
Aged 45–44	11 (17.7%)
Aged 55–64	14 (22.6%)
Aged 65+	26 (41.9%)
*Ethnicity*	
White British	55 (88.7%)
Asian British	2 (3.2%)
Black British	2 (3.2%)
Other	3 (4.8%)

61 participants (24 male and 37 female) completed ICECAP-A based on vignettes A, B and D. 53 participants completed ICECAP-A on behalf of person C (visual impairment)–this number excludes participants from the pilot workshop who saw a different vignette ‘C’.

The mean (self-reported) ICECAP-A score for participants (n = 62) was 0.8802 (SD: 0.1444, minimum: 0.2695, maximum: 1.0).

### Proxy ICECAP-A scores

Tables 2 and 3 show that mean proxy scores differ across the four vignettes, with the highest ICECAP-A score being associated with person B (planned pregnancy) and the lowest score being associated with person A (young adult feeling depressed). No statistically significant difference was found between the mean scores of male and female participants, either for own scores or for proxy scores across the four vignettes (Table 2). With respect to age, there were no statistically significant differences between means as determined by one-way ANOVA (Table 3). No correlation was observed between own ICECAP-A scores and proxy scores (for any of the four vignettes) (see Supporting Information).

**Table 2:** Proxy ICECAP-A scores by sex.

Vignette:	Mean ICECAP-A Score (Standard Deviation)			Two-tailed *p*
	Whole sample	Male participants	Female participants	
A (Depression)	0.3366 (n = 61) (SD: 0.1335)	0.3605 (n = 24) (SD: 0.1679)	0.3212 (n = 37) (SD: 0.1053)	0.3127
B (Planned Pregnancy)	0.9159 (n = 61) (SD: 0.0977)	0.8915 (n = 24) (SD: 0.1366)	0.9317 (n = 37) (SD: 0.0577)	0.1825
C (Visual Impairment)	0.8036 (n = 53) (SD: 0.1125)	0.7647 (n = 22) (SD: 0.1498)	0.8312 (n = 31) (SD: 0.0659)	0.0611
D (Dementia)	0.4292 (n = 61) (SD: 0.1583)	0.4343 (n = 24) (SD: 0.1665)	0.4259 (n = 37) (SD: 0.1550)	0.8416

**Table 3:** Proxy ICECAP-A scores by age.

Vignette:	Mean ICECAP-A Score (Standard Deviation)				*p*-Value
	Whole sample	Participants aged 18–44	Participants aged 45–64	Participants aged 65+	
A (Depression)	0.3366 (n = 61) (SD: 0.1335)	0.3188 (n = 10) (0.1124)	0.3306 (n = 25) (0.1410)	0.3493 (n = 26) (0.1372)	0.798
B (Planned Pregnancy)	0.9159 (n = 61) (SD: 0.0977)	0.9120 (n = 10) (0.0566)	0.9242 (n = 25) (0.0952)	0.9095 (n = 26) (0.1137)	0.862
C (Visual Impairment)	0.8036 (n = 53) (SD: 0.1125)	0.7548 (n = 9) (0.1517)	0.8447 (n = 23) (0.0685)	0.7795 (n = 21) (0.1222)	0.054
D (Dementia)	0.4292 (n = 61) (SD: 0.1583)	0.4608 (n = 10) (0.1088)	0.4266 (n = 25) (0.1695)	0.4197 (n = 26) (0.1666)	0.785

Standard deviation for the proxy scores is similar to that for the participants’ own scores. Proxy scores for vignette D ranged from zero to 0.9308. Proxy scores for Vignette C had the lowest range (0.4235 to 0.9456), but also the lowest number of observations.

### Reactions of participants to the vignettes

Many participants spoke of how they recognised or identified with aspects of the vignette; this was either through their own (direct) personal experience, through the experiences of people in their family and social networks, or through professional experiences. In the quotes below, ‘VA’, ‘VB’, ‘VC’ or ‘VD’ is used to denote the vignette being discussed in that specific section of the transcript.

*…when I … finished university I was pretty much in that exact situation myself*, *to be honest*: *Isolation*… (H01, M, 25–34) VA

*… can I just say*, *as a dad of someone who is like A*, *… my son*, *who was really depressed like this…* (A010, M, 45–54) VA

*We’re talking about ‘them’*, *who’ve got dementia*, *but we’re all secretly thinking “it could be us*.*” I know I am*, *because my mum and my grandmother had it*. (F03, F, 55–64) VD

It was common for participants to ‘bring the vignettes to life’; many participants assigned gender to the people in the vignettes.

*… you can be completely lonely in a big room of people*, *and that’s how he feels*. *(F08*, *F*, *65+)* VA

*She’s deeply unhappy*. *Clearly*. *She’s deeply unhappy*. (G05, M, 45–54) VA

There were, however, participants who reported that they were not able to identify with the people described in the vignettes and who expressed a lack of sympathy.

*…I don’t get a lot of sympathy for him*. *I’m just comparing him to myself*, *when I was nineteen*. *… and I just can’t- I can’t put myself in his position*. (F02, M, 65+) (VA)

*…he feels isolated*, *which to me that feels like that’s his own doing*. *Kick up the ass ‘pull yourself together and get on with life’* (D07, M, 55–64) (VA)

There was evidence that participants carefully read and considered the precise wording of the vignettes.

*I guess it does say ‘becoming’ isolated*, *not ‘have become’ isolated* (F03, F, 55–64) VA

### Approaches adopted by participants when providing proxy responses

Two themes were identified from the qualitative analysis which give an indication of how participants approached the task of providing proxy responses: they were interpreted as a factual approach (influenced either by a positive or negative interpretation of the facts) and an empathetic approach (aiming to respond as the subject would). A third, seemingly less common, approach involved participants drawing on their own related experiences, such that the subject of the vignette was no longer the participant’s sole focus and the proxy responses became more of an average response for people who might be in a similar type of situation.

#### Factual: Positive lens

This approach involved selecting facts that were relevant to the attribute/question, from a third-party perspective, and matching those facts to a response level. When participants shared their response with the group they specified which details from the vignette they had drawn upon. However, in arriving at a particular conclusion, participants appear to have filtered the facts according to their own sense of optimism, or focused in particular on positive aspects of the vignette. The terminology and description used in the vignette very much formed the core of what was said by participants, but to varying degrees there was also some embellishment of the detail or terminology used in the original vignette, which typically meant presenting facts in a more personable way.

*…they’re not socially isolated*, *they’re living with their parents and family*. *They’ve got friends*, *they are in a job so they’re not terribly sort of isolated*, *they have avenues they can express themselves with* (H07, 65+, M) VA

*…they’re reasonably prosperous*, *they’re in good- reasonably good health*, *despite the fags and being a bit overweight*. *They’ve got jobs and they’re*, *y’know*, *they’ve got a roof over their head and they’re financially fine*. *So*, *I can’t see anything that’d take them down from a four*… (H02, 55–64, M) VB

*… it says that this person is in contact with his son… on a really regular basis actually*, *and he also has a neighbour that will pop in if there is extra concerns*. (E007, 45–54, F) VD

#### Factual: Negative lens

Again, participants principally drew from the facts presented in the vignette, but this time either focused on negative aspects of the vignette or introduced an element of pessimism. And again, whilst participants used terminology that was closely aligned to the initial description, there was also some embellishment of the terminology. In the case of those viewing the facts more negatively (and linking to a sense of pessimism), embellishment also extended to what might potentially happen in the future (as illustrated by the second quote).

*… she hasn’t got a place of her own*, *she’s worrying about her weight [and] trying to tackle cigarettes*, *so I put 2*: *she’s secure in some areas*, *but I don’t think she is secure in all of them* (A012, 65+, F) VB

*… she’s not completely independent of her parents*, *she’s sort of dependent on them and sometimes that can cause issues*. *There’s a lack of option[s] there*… *You actually wouldn’t be able to afford to live anywhere else… if those circumstances change*… *(F05*, *55–64*, *M)* VB

#### Empathetic approach: Adopting the subject’s perspective

Although some participants did not explicitly suggest that they were responding from the perspective of the person in the vignette (the ‘subject’), they did seem to pick up a sense of the person’s attitude and reflect that back in their discussion (as illustrated in the first quotation). Other participants did explicitly state that they had responded as they thought the subject would respond.

…this person—like you said—must be a very positive person, although this is awful and it’s happened to them and they can’t see, they are still able to grab life with two hands and kind of still want to go to work and enjoy their life … (E02, 18–24, F) VC

… I put one again, because I don’t feel that this person—even if it is there–THEY don’t feel it [love, friendship and support] (D001, 65+, F) VA

There were examples of when participants reported having given a response from the subject’s perspective which they perhaps didn’t agree with themselves objectively. Participants sometimes reported that they were unsure which perspective they were being expected to adopt, or were perhaps torn as to which perspective they should adopt.

*I think [level] one was exactly where they would be in their mind*, *whether-*, *looking from an outside point of view*, *…a lot of them could probably be three or four*, *but from their inside point of view I’d say one-* (H01, 25–34, M) VA

*… I would imagine she herself would put one in all cases*, *but if we were filling it out for her or helping her we might think it’s actually a two there*. (G05 45–54, M) VA

*This is a question*, *with the [Dementia vignette] they mostly think they’re independent*, *they can do a lot of things*, *but in fact they don’t*. *So*, *what do you put it down as*? (H06, 35–44, F) VD

#### Drawing upon external experiences/references

Participants occasionally introduced external experiences which appeared to strongly influence their response, perhaps outweighing detail from the vignette.

*…well I put 3*. *I put that because although they are depressed they don’t stop loving their family*. *A friend of mine*, *her son just committed suicide and I know that he loved-*, *its mental state*, *but he still loved them*, *that’s why I put that* (A12, 65+, F) VA

*I just think they have to wait for the baby to be born ‘cause I know how I felt when I was pregnant and*, *you just worry constantly until the baby’s born* (H04, 45–54, F) VB

#### Interpretation of concepts from ICECAP-A

There was debate in some groups around the meaning of the term independence (the autonomy attribute), with different interpretations emerging. Two issues in particular sparked debate: financial independence (in this case, relating to home ownership), and the independence of a person with family commitments (i.e. the issue of combined capability).

*Living in maybe- something about living in a rented flat*. *I mean, perhaps they can’t afford to buy their own house yet*. (*G05, 45–54, M*)

• *That’s a very British attitude though, isn’t it?* ‘*Cause in Germany most people rent their homes and they’re perfectly fine with that*. (*G06, 55–64, F*)

*… if you’re actually married and in a relationship it’s impossible to be completely independent because you have to surrender a certain amount to your partner*, *don’t you*? (H02, 55–64, M)

*…I don’t think any of us are completely independent* (B04, 45–54, F)

In relation to the Achievement attribute on ICECAP-A, there was evidence that participants’ ideas of what constituted achievement were context (including life-stage and health) specific. There was a focus on job security and career progression for vignettes B and C, and on the person in Vignette A going to university. An achievement for person D was living independently.

*She’s got a management job as well*, *it may only be assistant manager*, *but she has to make decisions and leadership at certain levels*. (B05, 55–64, F) VB

*And it sounds like they can further progress in their working life*, *because the-*, *the company is supporting them*. *(F09*, *45–54*, *M)* VC

*…but I think she is still able to achieve a lot at the moment*, *because she can still go out and go to the shop—although she might forget what she’s gone for–she’s still able to get out there …*. (C05, 65+, F) VD

## Discussion

Much of the literature on proxy completion has focused on the assessment of health related functioning and on the correlation between patient and proxy responses. The ICECAP measures assess well-being in a broader sense and rely on the potentially complex concept of capability (a distinction between functioning and the ability to function). There is some evidence of differences between proxy assessments of well-being by care workers and proxy assessments of well-being by family members for care home residents, when assessed using ICECAP-O [13] and some evidence that health professionals find proxy completion of EQ-5D easier than proxy completion of ICECAP-A in the context of care at the end of life [14].

This study has adopted the novel approach of eliciting multiple proxy responses for a defined set of hypothetical vignettes. The study found no evidence that participants’ own demographic or well-being characteristics systematically influenced proxy responses, but did find that there were large ranges of values for the same individual/vignette. Variation across proxy respondents for the same vignette appears to have been driven by four factors: the perspective adopted (factual versus empathetic); the proxy’s ability to empathise with the subject; the introduction of external information and considerations; the proxy’s interpretation of the measure attributes.

Participants differed in their attitudes to mental health problems, with some reporting that they were unable to empathise or sympathise in particular with the individual described in vignette A (depression). The range of scores for scenario D indicate that proxy respondents struggled when the individual described in the vignette had a cognitive impairment (in this case, dementia). Considering that ICECAP-A scores are anchored between zero and one, proxy scores for vignette D ranged massively (from zero to 0.9308).

In the case of cognitive impairment, there may be significant differences in scores depending upon whether the proxy respondent adopts a factual or an empathetic approach. The two approaches identified here (factual and empathetic) closely relate to and can be retrospectively matched to Pickard and Knight’s two perspectives: proxy-proxy and proxy-patient [16]. We haven’t attempted to explore potential differences in ICECAP-A scores from proxy respondents adopting the two different approaches (factual versus empathetic) because it cannot be guaranteed that participants adopted the same approach consistently across either all four vignettes or across all five questions from the ICECAP-A. It would also not be possible to identify the approach that was adopted by all of the participants–some articulated their approach more explicitly than others.

A limitation of the study is that there was no professional or personal relationship between proxy respondents and subjects, although this fact did not result in participants being noticeably cautious with their proxy responses–participants did not, for example, avoid extreme response levels. The lack of sympathy or empathy for the individuals described in the vignettes (observed in a small number of cases) would perhaps be less likely to be observed in cases where there was a personal relationship between the proxy and the subject. It may be that participants in this study were more prone to introduce additional information into their assessment (as evidenced through the qualitative analysis) because of the lack of a relationship with the subject and perhaps, linked to this, the fact that the vignettes were deliberately fairly brief. The introduction of considerations or reference points which were external to the subject of interest by participants in our study is a phenomenon which Makai *et al* suspected having occurred in their study; they suggested that care staff in the nursing home context could have been drawing upon experiences of other residents as a point of reference when providing proxy responses [13]. The naming of health conditions or types of disability (for example, using the term ‘dementia’) may have encouraged the introduction by participants of external knowledge and points of reference, but it is reasonable to expect that when providing proxy responses for an actual close person, the proxy rater would be aware of their health constraints and diagnoses. Previous quantitative research has found that labelling health states influences health state valuation [19, 20]; clearly the context and methodologies differ, but the qualitative evidence from this study of proxy raters introducing external considerations relating to a particular illness/diagnosis may be relevant to those undertaking future research involving health state valuation and choosing whether to label health states.

Given the broad scope of the concepts covered by ICECAP-A it is likely that, even where there is a close personal or professional relationship with the subject, proxy responses will be determined to some extent by the proxy’s own interpretation of the attributes. In particular, there was discussion and some disagreement amongst participants in this study over the interpretation of autonomy (independence) and achievement. Participants differed, for example, in the extent to which they considered finances (including home ownership) as an element of independence, and in the extent to which they considered family as a constraint to personal independence. There was some evidence that participants had different expectations of what constituted achievement, given the life-stage of the individual described in the vignette.

Because vignettes were developed depicting hypothetical individuals, it was not possible to compare proxy responses with responses from self-completion, although doing so was not the aim of this particular study.

A limitation of the study was the sample size. Although no other study, to our knowledge, has elicited or been able to compare as many as 61 different proxy responses for the same individual, the sample size was only sufficient to enable somewhat tentative quantitative analysis.

## Conclusion

This study adds to the existing body of evidence suggesting that proxy completion is problematic. Given the scope for different proxy respondents to differ in relation to both perspective (empathetic, linking to Pickard and Knight’s ‘proxy-patient’ perspective versus factual, linking to the proxy-proxy perspective) and interpretation of concepts covered by the questions, at the very least the same questions should be asked of the same proxy respondent at each point of data collection. Relying on a single proxy respondent will ensure that variation in responses/scores across different time points is driven by a proxy respondent’s perception of variation in the subject’s well-being, and not by differences in how proxy respondents approach the task of providing proxy responses.

It may be worth explicitly instructing proxy respondents to adopt either a proxy-patient (empathetic) or a proxy-proxy (factual) perspective, as per Pickard and Knight’s recommendation, although it is not clear to what extent proxy respondents will take note of or adhere to this instruction (either at all or consistently across different questions). It may also be worth selecting proxy respondents who are homogenous in terms of the nature of their relationship with the subject, for example, all proxy respondents will be health/care professionals or all will be family/friends. Homogeneity in proxy respondents may promote homogeneity in the type of external information that is potentially introduced by proxies as their point of reference.

## Supporting information

S1 Appendix (DOCX)

S1 Proxy data (XLSX)

S1 Table (DOCX)
