# Supplementary material for: Proxy responses to ICECAP-A: Exploring variation across multiple proxy assessments of capability well-being for the same individuals
Source: PLoS One. 2020 Jul 28;15(7):e0236584. doi: 10.1371/journal.pone.0236584 (PMC7386591; doi:10.1371/journal.pone.0236584)
Supplement: S1 Appendix — (DOCX) [file pone.0236584.s001.docx]

**S1 Appendix**

**Proxy responses to ICECAP-A: Exploring variation across multiple proxy assessments of capability well-being for the same individuals**

Philip Kinghorn & Nafsika Afentou

**Person A**

A 19 year old who lives at home with their parents and younger sibling.

Person A has been feeling depressed for 18 months now. There are days when they wish that they were dead. They are confused as to where to turn to for support and have not yet discussed the problem with their GP or parents. Given that they look healthy ‘on the outside’ they are struggling to think of how they can tell people that there is something wrong. They worry that since leaving college they are becoming isolated. Their anxiety and depression has had an impact on their eating habits and they have started to lose weight.

They see their friends less frequently now and when they do, their friends have noticed that they drink heavily.

They have delayed going to university and are working part-time in a fast food outlet.

**Person B**

A 29 year old who has just had confirmation from their GP that they are pregnant with their first child. The pregnancy was planned and both person B and their partner are feeling excited. It is a happy and stable relationship.

They are currently a smoker and have been since the age of 16. They have managed to cut the number of cigarettes that they smoke a day down to four, but are seeking help to stop completely.

They do not exercise regularly and are slightly overweight, but do not have any health problems at the moment. Their partner does not smoke and enjoys good health.

They work as an assistant manager at a supermarket. The couple live in a flat owned by person B’s parents, and pay reduced rent.

The couple have an established group of friends and modest financial savings.

**Person C**

A 47 year old who has been severely visually impaired for the last 6 years.

They are married with a 15 year old daughter. Their partner undertakes many roles around the house, such as cooking, cleaning and reading bills and letters. Their partner helps them with some aspects of personal care, such as fastening buttons and turning on the shower.

They have a severely reduced ability to make out detail from across a room and have a reduced field of vision (they can see very little from the side of their eye when looking straight ahead). Detail that they would struggle to see across a distance equal to an average sized room would include: kerbs, pay points in shops, people, and small to medium sized items of furniture. They are unable to use a standard mobile phone.

They work as a programme manager for a national charity. They rely on their partner to drive them to and from work. Whilst at work they use computer software to read text, bought for them by their employer. They use a white cane.

They find their job rewarding and have a permanent contract. They enjoy listening to the radio and walks with the family.

It is possible that their vision will worsen in the future.

**Person D**

A 68 year old with dementia. They live alone in their council house.

They have one son, who lives 45 minutes away and visits as often as he can (one to three times per week). A neighbour will call in to check on Person D if they have concerns.

Their dementia means that although they can do many things for them self, they do have problems with short term memory and taking in new information. At this stage, their forgetfulness is mainly an inconvenience, but could potentially be dangerous. They do miss some meals. They can struggle to find things that they need when in their own home and can become frustrated.

They walk the short distance to the local shop and GP surgery, but can become lost if things change along the route. They do forget what items they need when they get to the shop and seek help from staff to find where products are located. Their son rings to remind them of appointments. Prescriptions are delivered to the home, with medication sorted into days of the week.

They spend much of their time watching TV. They now find it difficult to concentrate on jigsaw puzzles and have largely stopped attempting these after experiencing increasing frustration. Much of the time they are socially isolated.
