# Supplementary material for: Proxy responses to ICECAP-A: Exploring variation across multiple proxy assessments of capability well-being for the same individuals
Source: PLoS One. 2020 Jul 28;15(7):e0236584. doi: 10.1371/journal.pone.0236584 (PMC7386591; doi:10.1371/journal.pone.0236584)
Supplement: S1 Table — (DOCX) [file pone.0236584.s003.docx]

**S1 Table**

**Proxy responses to ICECAP-A: Exploring variation across multiple proxy assessments of capability well-being for the same individuals**

**Philip Kinghorn & Nafsika Afentou**

**Plotting Participants’ own ICECAP scores against their proxy scores:**
